# Inhibition of BMP signaling pathway induced senescence and calcification in anaplastic meningioma

**DOI:** 10.1007/s11060-024-04625-2

**Published:** 2024-03-06

**Authors:** Kiyotaka Yokogami, Takashi Watanabe, Shinji Yamashita, Asako Mizuguchi, Hideo Takeshima

**Affiliations:** Department of Neurosurgery, Faculty of Medicine, University of Miyazaki, Miyazaki, 889-1692 Japan

**Keywords:** Malignant meningioma, Induced calcification, Senescence, BMP, GREM2

## Abstract

**Purpose:**

Meningiomas are the most common type of brain tumors and are generally benign, but malignant atypical meningiomas and anaplastic meningiomas frequently recur with poor prognosis. The metabolism of meningiomas is little known, so few effective treatment options other than surgery and radiation are available, and the targets for treatment of recurrence are not well defined. The Aim of this paper is to find the therapeutic target.

**Methods:**

The effects of bone morphogenetic protein (BMP) signal inhibitor (K02288) and upstream regulator Gremlin2 (GREM2) on meningioma’s growth and senescence were examined. In brief, we examined as follows: 1) Proliferation assay by inhibiting BMP signaling. 2) Comprehensive analysis of forced expression GREM2.3) Correlation between GREM2 mRNA expression and proliferation marker in 87 of our clinical samples. 4) Enrichment analysis between GREM2 high/low expressed groups using RNA-seq data (42 cases) from the public database GREIN. 5) Changes in metabolites and senescence markers associated with BMP signal suppression.

**Results:**

Inhibitors of BMP receptor (BMPR1A) and forced expression of GREM2 shifted tryptophan metabolism from kynurenine/quinolinic acid production to serotonin production in malignant meningiomas, reduced NAD + /NADH production, decreased gene cluster expression involved in oxidative phosphorylation, and caused decrease in ATP. Finally, malignant meningiomas underwent cellular senescence, decreased proliferation, and eventually formed psammoma bodies. Reanalyzed RNA-seq data of clinical samples obtained from GREIN showed that increased expression of GREM2 decreased the expression of genes involved in oxidative phosphorylation, similar to our experimental results.

**Conclusions:**

The GREM2-BMPR1A-tryptophan metabolic pathway in meningiomas is a potential new therapeutic target.

**Supplementary Information:**

The online version contains supplementary material available at 10.1007/s11060-024-04625-2.

## Introduction

Meningiomas are brain tumors that account for one-third of all CNS tumors, arise from arachnoid cells and dural border cells tightly adherent to the dura [1, 2], and affect the surrounding bone in 25% to 50% of cases, causing bone changes such as hyperostosis and ballooning [1, 2]. Calcification can also occur within the tumor, and tumors with calcification often do not enlarge [3]. Malignant atypical meningiomas and anaplastic meningiomas frequently recur and exhibit aggressive behavior with or without bone destruction [4], resulting in 5-year survival rates of 67.5% for atypical meningioma and 55.6% for anaplastic meningioma. No effective chemotherapy is available for meningioma, and incomplete resection and irradiation of < 50 Gy significantly reduces the 5-year progression-free survival rate compared with complete resection followed by irradiation of 50-62 Gy [4]. The survival benefit of boron neutron capture therapy, a novel radiation therapy, is approximately 2 years [5]. Consequently, the indications for reoperation and irradiation are limited for recurrent anaplastic meningiomas [6, 7], so new treatment strategies are needed.

Meningiomas are closely associated with ossification and calcification, and the involvement of bone morphogenetic protein (BMP), insulin-like growth factor-1, endothelin-1, and osteoprotegerin has been investigated, but any function in meningioma cells is still unknown [8]. The new WHO 2021 classification scheme of meningiomas added gene and methylation profiles which are strongly correlated with the prognosis [9–13], but how changes in these genes and methylation profiles relate to meningioma metabolism and drug treatment targets remain unclear.

This study investigated the involvement of Gremlin2 (GREM2), an inhibitor of BMP signaling, particularly the BMP signaling essential for bone formation, in meningioma growth, senescence, and calcification via tryptophan metabolism. Our findings show that the metabolic characteristics and phenotype of benign and malignant meningiomas depend on BMP signaling and represent a new drug therapeutic target for atypical meningioma and anaplastic meningioma.

## Methods

### Cell lines, Culture conditions and surgical samples

Human meningioma cells (MZ821M, MZ840M, MZ842M, and MZ857M) were isolated and established as cell lines from the surgical tissues of patients treated in our institution from 1st April 2019 to 31 March, 2022 (Supplementary Table 1). Meningioma cells were cultured using conditional reprogramming methods [14] on collagen-coated 10-cm culture dishes in a humidified incubator at 37 °C under an atmosphere of 5% CO_2_ and 95% air. HKBMM and IOMM-Lee were cultured with a standard method. K02288 was purchased from Selleck (Tokyo, Japan). Eighty-seven frozen samples (Grade 1: 63, Grade 2: 18, Grade 3: 6) of meningioma cases operated on at our hospital between 2016.3 and 2022.3 were used for the cohort analysis of mRNA expression and MIB-1 labeling index (MIB-1 L.I.) of surgical specimens.

### Plasmid preparation and transfection

Wild type human GREM2 was synthesized (Thermo Fisher Scientific, Waltham, MA, USA) and cloned into pcDNA3.1. Empty vector or pcDNA3.1-GREM2 was transfected into malignant meningiomas (IOMM-lee, HKBMM, and MZ857M) by electroporation. Neomycin was used for selection of stably transfected cells.

### Alizarin red s staining

Meningioma cells were seeded in 6-well plates at a concentration of 2 × 10^5^/ml. When the meningioma cells were 70% confluent, the meningioma cells were divided into two to three wells. K02288 were added to the standard medium for 30 days. During staining, cells were washed twice with PBS, fixed with 4% paraformaldehyde at 4 °C for 10 min, washed 3 times with deionized water, washed with 1% Alizarin Red (Sigma-Aldrich, St. Louis, MO, USA), and incubated at room temperature for 20 min. PBS was used to remove excess dye.

### RNA extraction, cDNA synthesis, and quantitative polymerase chain reaction (qPCR) analysis

RNA from cultured tumor cells was extracted with RNeasy Plus Mini Kit (QIAGEN, Germantown, MD, USA). For cDNA synthesis, RNA was reverse transcribed from random hexamers using the SuperScript™ VILO™ cDNA Synthesis Kit (Invitrogen, Life Technologies, Thermo Fisher Scientific, Waltham, MA, USA). Real-time qPCR was then performed in triplicate on the StepOne Plus or Quant3 (Applied Biosystems, Thermo Fisher Scientific) using SYBR™ Green Realtime PCR Master Mix (Applied Biosystems, Thermo Fisher Scientific) to determine the mRNA levels. PCR was performed using a 20 μl volume containing 2 µl cDNA, 300 µM of each primer, and 10 µl of 2 × PCR master mix under the following conditions: 95 °C for 10 min followed by 40 cycles of 95 °C for 15 s and annealing/extension at 60 °C for 1 min. The data were normalized to the amount of human 18S rRNA, and the values are represented as the mean ± standard deviation of 2 − ΔΔCt in a triplicate assay. Primer sequences were GREM2-Fw: atcccctcgccttacssgga and GREM2-R: tcttgcaccagtcactcttga.

### In silico expression analysis

Gene expression data (GSE101638) obtained by the RNA-sequencing technique were downloaded from the public database GEO RNA-seq Experiments Interactive Navigator (GREIN) (http://www.ilincs.org/apps/grein/?gse =). The average of GREM2 gene expression levels of 42 samples in dataset GSE101638 was calculated, and 10 cases with higher levels were defined as GREM2-high and 32 cases with lower levels as GREM2-low. The data from the GeneChip™ System described above and downloaded data were analyzed with gene set enrichment analysis (GSEA) (http://www.broad.mit.edu/GSEA) using MSigDB (v2023) to determine the significance of a pre-defined gene set by comparing the correlation between expression and class distinction with other random situations. The significance threshold was set at nominal *P* value < 0.05 or false discovery rate q value < 0.25.

### Cell cycle analysis

The Cell Cycle Phase Determination Kit (Cayman Chemical, Ann Arbor, MI, USA) was used for cell cycle analysis. In brief, collected cells were rinsed twice with buffer, then fixed at − 20 °C overnight. Cells were washed twice with ice-cold PBS, and stained with propidium iodide/RNase staining buffer solution in the dark for 30 min at room temperature. Then, cells were analyzed with a flow cytometer (Guava® EasyCyte™ Mini; Luminex Japan, Tokyo, Japan). A histogram of the cell cycle distribution was generated from 5000 events per sample and data were analyzed using Guava® Cell Cycle software.

### ATP and NAD/NADH assay

ATP assay kit-luminescence (A550) and NAD/NADH assay kit-WST (N509; Dojindo, Kumamoto, Japan) were used to measure the intracellular ATP/NAD/NADH following the manufacturer’s instructions. A Beckman Coulter DTX-800 multimode detector with multimode analysis software platform (Beckman Coulter, Brea, CA, USA) was used to calculate the ATP levels. For NAD/NADH measurement, the plates were read at 450 nm.

### ELISA for serotonin measurement

The meningioma cells with/without 30 µM of K02288 for 72 h were homogenized by sonication in ice-cold PBS followed by centrifugation at 10,000 g for 5 min. Intracellular serotonin was measured using the serotonin ELISA Kit (KA2518; Abnova, Taipei, Taiwan) according to the manufacturer's instructions under the indicated conditions. The standards were generated using the serotonin standard supplied in the kit. After the reaction was stopped, the optical densities at 450 nm (OD450) and at 620 nm (OD620) were read using a SpectraMax iD3 Multi-Mode Microplate Reader (Molecular Devices, San Jose, CA, USA).

### SPiDER-βGal staining

The meningioma cells were cultured with/without 30 µM of K02288 for 5 days. The cells were washed with 2 ml of Hanks’ HEPES buffer twice. SPiDER-βGal (SG02; Dojindo) working solution (1 μmol/l) was added to the culture dish, and the cells were incubated for 15 min at 37 °C. The dye was washed off and observed under a fluorescence microscope.

## Results

### Inhibition of BMP signaling reduced proliferation. Moreover, long-term inhibition of BMP signals induced calcification

**Fig. 1:**
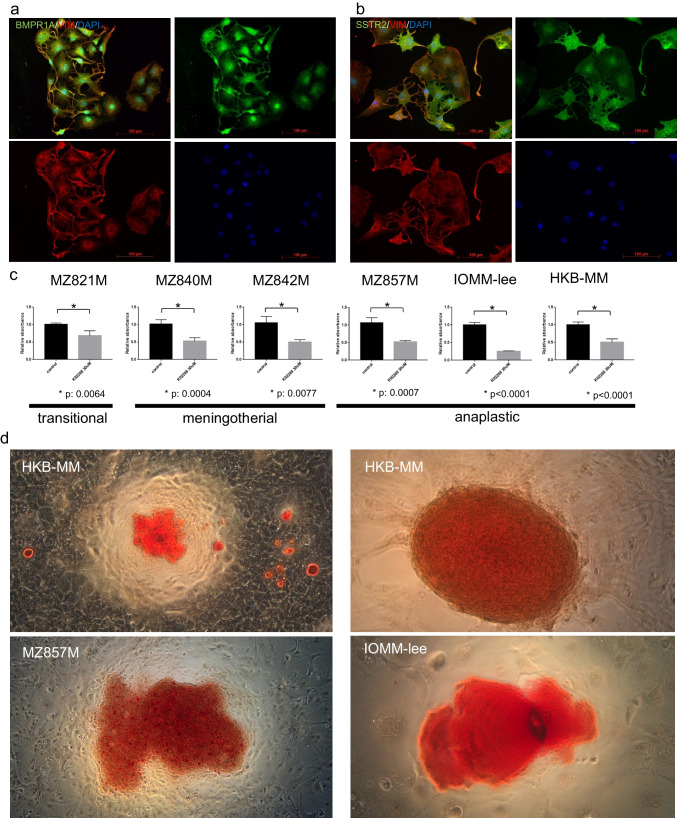
BMP signaling pathway involved with proliferation and induced calcification. Three characteristic shapes of tumor cells can be seen in this culture: 1) Small cytoplasm with bilaterally extended projections, 2) Small cytoplasm with numerous spinous processes, and 3) Large cytoplasm without spinous process making pavement pattern. The tumor cells are positive (*green*) for the BMP receptor BMPR1A (**a**). The first two shapes are strongly positive (*green*) for SSTR2, but the last one is weekly positive for SSTR2 (**b**). Chemical inhibition by K02288 decreased the growth rate regardless of grade, indicating that the BMP signal is involved in the growth of meningiomas in general (**c**). Long-term culture for 1 month with K02288 induced calcification in anaplastic meningioma cell lines MZ857M, HKBMM, and IOMM-Lee (**d**). Low (*left*) and high magnification (*right*)

Primary culture was performed using the conditional reprogramming method. Anaplastic meningioma MZ857M could be passaged for more than 2 years. Primary cultured meningioma cells showed very characteristic morphology and expressed the BMP receptor (BMPR1A) (Fig. 1a). SSTR2 was strongly positive for cells with a numerous spinous process and weakly positive for cells with large cytoplasm and no spinous process (Fig. 1b). Application of the BMP inhibitor K02288 to primary cultured cells from 3 cases of WHO grade 1, 1 case of grade 3, and 2 cases of commercially available cell line grade 3 showed K02288 inhibited growth regardless of grade (Fig. 1c). The concentration of the IC_50_ suggested that BMPR1A was involved (Supplementary Fig. 1) [15]. Surprisingly, after 1 month of treatment of anaplastic meningioma with K02288, not only growth inhibition but also calcification of the tumor cells was observed (Fig. 1d). This calcification consisted of a black cellular residue-like structure in the center, surrounded by calcium deposits.

### Expression and localization of GREM2 in surgical samples

**Fig. 2:**
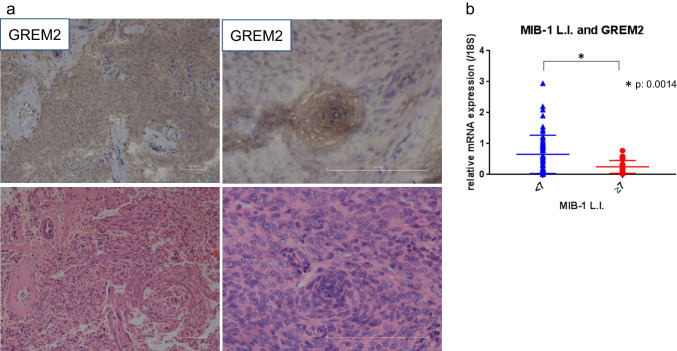
(**a**) Expression of GREM2, upstream regulator of the BMP signal, related to the proliferation index. GREM2 was stained diffusely in some tumors and partially in others, and did not correlate with histological type. Cells that were partially stained were well stained in the whorl formation. (**b**) Given that MIB-1 labeling index is associated with meningioma recurrence, as > 7 is associated with a significantly higher risk of recurrence,^5^ so the association between MIB-1 labeling index and GREM2 was investigated in 87 cases (> 7: 27 cases, < 7: 60 cases). The Figure shows a significant correlation between MIB-1 labeling index and GREM2 mRNA expression

Although benign and malignant cells have different proliferative capacities, K02288 had the same level of growth inhibition effect in both benign and malignant cells. Therefore, we hypothesized that upstream regulators that control BMP signaling may be differentially expressed between benign and malignant cells. GREM2 was identified as an upstream regulator of BMP signals in a PubMed keyword search and meningiomas [16]. GREM2 expression and localization were examined in clinical samples, and GREM2 was found to be almost completely absent in grade 2.3 meningioma by immunostaining, but was strongly expressed around psammoma bodies and at sites of whorl formation grade 1 meningiomas (Fig. 2a). Since MIB-1 labeling index > 7 is reported to have significantly higher recurrence [17], we compared GREM2 expression between the two groups of MIB-1 labeling index > 7 and < 7, and found that GREM2 expression was strongly correlated with the growth rate (MIB-1 labeling index) (Fig. 2b).

### Suppression of BMP signaling including overexpression of GREM2 increases senescence and inhibits cell proliferation

**Fig. 3:**
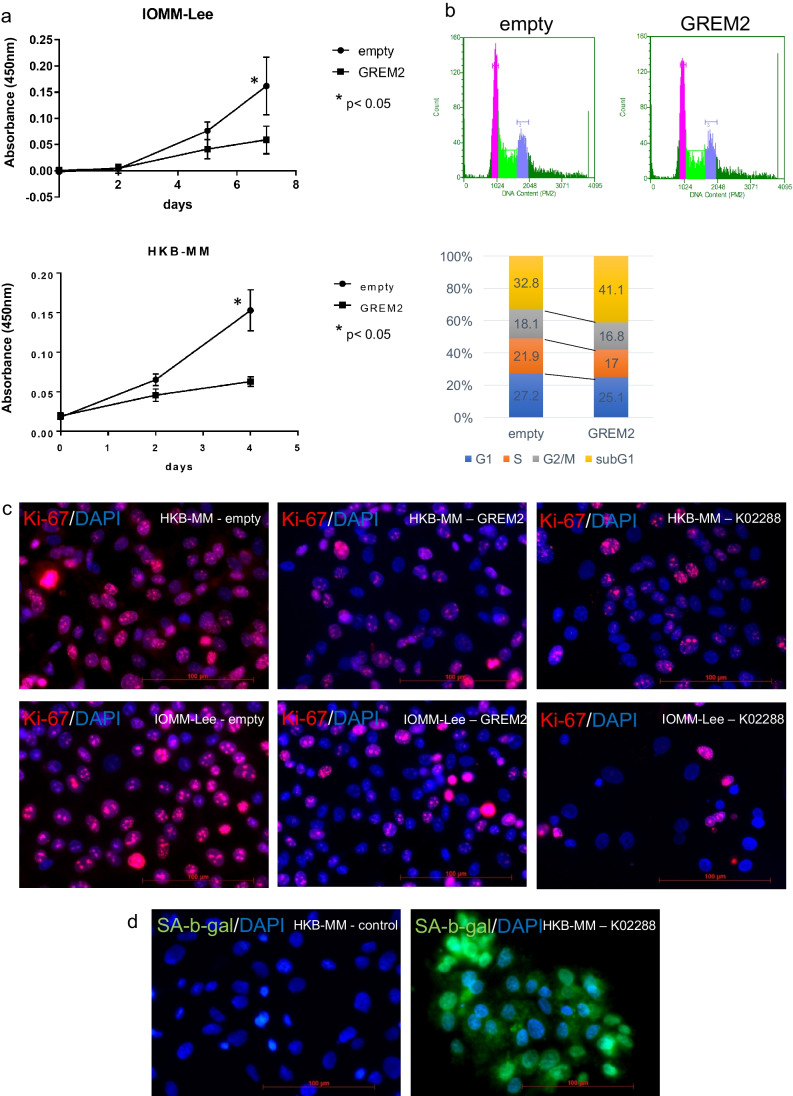
Overexpression of GREM2 reduces S phase population and inhibits cell proliferation. The GREM2 expression vector was created and overexpression experiments were performed using IOMM-Lee and HKBMM cell lines (not shown). The WST-8 assay shows GREM2 expression in IOMM-Lee inhibited proliferation (**a**), and cell cycle analysis indicated prominent reduced S-phase and increased sub-G1 cells (**b**). Overexpression of GREM2 or addition of K02288 reduced Ki-67, a marker of cellular senescence and proliferation in HKBMM and IOMM-Lee (**c**). Senescence-associated β-galactosidase (SA-β-gal) assay showed K02288 induced senescence (*green*) in HKBMM (d)

Forced expression of GREM2 in anaplastic meningioma (IOMM-Lee, HKBMM) and subsequent proliferation assay found that GREM2-expressing cells showed decreased proliferation rate (Fig. 3a). The cell cycle showed that GREM2 expression increased the number of sub-G1 cells (Fig. 3b). Based on cell cycle analysis, we thought that the cells might be in cellular senescence. Suppression of BMP signaling decreased Ki-67 staining (Fig. 3c) and increased β-galactosidase expression (Fig. 3d, Supplementary Fig. 3), proving that suppression of BMP signaling induced senescence.

### Expression of GREM2 increases suppressive 5-hydroxytryptamine receptors expression and shifts tryptophan catabolism from kynurenine/NAD/NADH to serotonin production

**Fig. 4:**
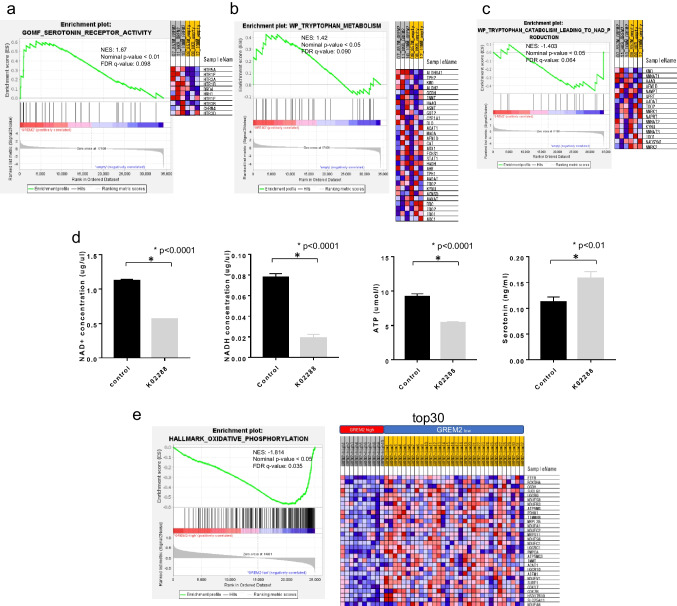
Enrichment analysis showed that GREM2 overexpression caused skew of tryptophan metabolism toward serotonin production from NAD + production. Overexpression of GREM2 increases the expression of inhibitory serotonin receptors (**a**) and enzymes involved in serotonin synthesis through tryptophan metabolism, and conversely decreases the expression of enzymes involved in kynurenine/quinolinic acid synthesis (**b**). Overexpression of GREM2 also decreases the expression of NAD + synthetase (NADSYN1) and other enzymes involved in NAD + production, as well as NMRK2 and other enzymes involved in the salvage pathway (**c**). Metabolites were measured by ELISA in HKBMM cell line cultured with/without K02288 for 3 days. BMP signal inhibition decreased NAD + /NADH/ATP, but increased serotonin production (**d**). Each experiment was performed in triplicate. GSE101638 dataset (RNA-sequencing data in public database) was re-analyzed with GSEA. Low GREM2 expression group had significantly higher gene expression for the oxidative phosphorylation pathway, which primarily uses NAD + to produce ATP (**e**)

We comprehensively analyzed which genes are affected to explain the change in proliferation rate and senescence induced by GREM2 expression. The following results were obtained: 1) GOMF_SEROTONIN_RECEPTOR_ACTIVITY (Fig. 4a) 2) WP_TRYPTOPHAN_METABOLISM (Fig. 4b) and 3) WP_TRYPTOPHAN_CATABOLISM_LEADING_TO_NAD_PRODUCTION (Fig. 4c).

Forced expression of GREM2, which represses BMP signaling, alters mRNA expression involved in tryptophan metabolism (Fig. 4b and c), decreases mRNA expression involved in NAD production (Fig. 4c), and increases mRNA expression involved in serotonin receptor activity (Fig. 4a). It is well known that the function of serotonin depends on its receptors, and HTR5A, HTR1F, and HTR1B in the Top5 are inhibitory receptors that decrease intracellular cyclic adenosine monophosphate (cAMP) (Fig. 4a). Next, to measure these final metabolites, we suppressed the BMP signal using K02288, which decreased NADH/NAD + /ATP and increased serotonin, reflecting the above results (Fig. 4d).

### Comprehensive analysis using RNA-sequencing data from GREIN showed that high expression of GREM2 decreases mRNA expression affected by oxidative phosphorylation and increases suppressive 5-hydroxytryptamine receptors

Since NADH/NAD + is an essential cofactor for oxidative phosphorylation, we examined whether the degree of GREM2 expression changed expression of the group of enzymes related to oxidative phosphorylation in the clinical samples (Fig. 4e). RNA-sequencing data from 42 meningiomas were obtained from the public database GREIN (GSE101638) and the mean expression level of GREM2 was calculated. Two groups of 32 cases with higher-than-average expression levels and 10 cases with lower-than-average expression levels were identified. Analysis of this data using the GSEA hallmark gene sets revealed significantly elevated expression of a group of enzymes related to oxidative phosphorylation in the group with low GREM2 expression (nominal p-value < 0.05, FDR q-value: 0.035). GOBP_SEROTONIN_RECEPTOR_SIGNALING_PATHWAY was also significantly upregulated in the high GREM2 expression group (nominal p-value < 0.01, FDR q-value: 0.021) (Supplementary Fig. 2).

## Discussion

**Fig. 5:**
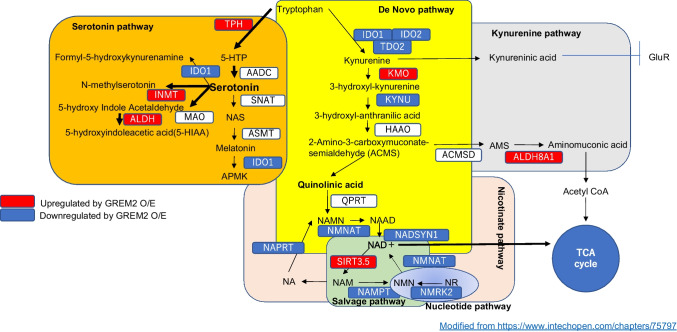
Schematic presentation of tryptophan metabolism. Genes upregulated by GREM2 overexpression are indicated by red colored squares and genes downregulated are indicated by blue colored squares. Overexpression of GREM2 shifts tryptophan metabolism from kynurenine/quinolinic acid to serotonin production

Meningiomas are tumors that cause thickening of the adjacent bone, and the tumors also contain calcifications such as psammoma bodies. Morphological observation of the granular body indicates that degenerated membrane-forming vesicle-like cellular debris (matrix vesicle) is located in the center of the whorl, from which the calcification begins and spreads to the surrounding area to form the granular body [18–20]. The mechanisms of calcification, degeneration by cellular senescence, and tumor growth may be closely related, since the psammomatous subtype of meningioma with strong calcification is known to proliferate at a slower rate and recurs less frequently, even after incomplete resection [21]. In particular, BMP signals, which are central in bone metabolism, may lead to meningioma development and adjacent bone changes and tumor growth, but few studies have considered this observation. Culture of four primary cases of WHO grade 1 meningiomas showed that BMP-4 promotes meningioma growth in an autocrine/paracrine pathway via Smad1 [9], and BMP-4 is highly expressed in osteolytic meningiomas [22], but BMP signaling inhibitors or their inhibitors are not known to be involved in cellular senescence or formation of psammoma bodies. Artificially induced calcification has not been attempted in malignant meningiomas in vitro. The present study reproduced the process of whorl formation around small calcifications and their transformation into large calcified lesions on culture dishes by controlling the BMP signals, regardless of the type of malignant meningioma (Fig. 1d), and showed that the detailed underlying mechanism is cellular senescence due to a shift in tryptophan metabolism (Fig. 5).

BMP signaling is initiated by the binding of the BMP ligand to the tetrameric receptor formed of two molecules of the two types of BMP receptor, type 1 and type 2 [23]. Type 1 receptors are BMPR1A (ALK3), BMPR1B, and ACVR1, and type 2 receptors are BMPR2, ActR2A, and ActR2B. Ligands BMP2 and 4 bind to BMPR1A, and ligands BMP6 and 7 to ACVR1 [24, 25]. GREM2 is a known BMP antagonist, and detailed studies, including crystallographic analysis, have revealed that GREM2 inhibits BMP signaling by forming a complex with growth/differentiation factor 5 and binding to BMPR1A [26, 27]. Our study of the inhibitory concentration of K02288 suggested that inhibition of BMPR1A occurred, and since GREM2 also inhibits BMPR1A, so we conclude that BMPR1A-mediated signals are responsible for the growth, cellular senescence, and calcification of meningiomas. GREM2 was identified as one of the factors involved in malignant transformation of meningiomas by bioinformatics analysis [16], but the mechanism of GREM2 expression leading to suppression of BMP, the transforming growth factor-β signaling pathway, and tumor progression, was unclear. Our present results showed that GREM2 was highly expressed in grade 1 low MIB-1 samples, similar to the previous findings and was barely expressed in grade 2 or 3 high MIB-1 samples. Moreover, strong GREM2 expression around the psammoma body of grade 1 meningiomas and at the sites of whorl formation suggested that the GREM2/BMP signal was strongly associated with tumor calcification and proliferative capacity.

To test this hypothesis, GREM2 was overexpressed in three strains of malignant meningiomas, and as expected, growth was suppressed. The cell cycle showed that both GREM2 overexpressing cells and BMP inhibitors resulted in increased sub-G1, suggesting that the cell cycle is inclined toward cellular senescence. We investigated the detailed mechanism by comprehensive expression analysis using microarrays and found that GREM2 overexpression suppressed BMP signaling by 1) increasing the expression of inhibitory serotonin receptors, and 2) decreasing the expression of enzymes involved in the production of kynurenine/NAD + /NADH and increased expression of enzymes involved in the production of serotonin in the tryptophan metabolic pathway. Consequently, NADH and NAD + were decreased and serotonin was increased. The effects of serotonin are mainly determined by the nature of its receptors, and an increase in serotonin receptors of the present inhibitory system decreases intracellular cAMP [28].

NAD + is present in all living cells, and functions as an important redox cofactor for enzymes that facilitate reduction and oxidation reactions by transporting electrons from one reaction to another, as a co-substrate for other enzymes such as sirtuins and polyadenosine diphosphate ribose polymerase, and is central in redox reactions [29]. Decreased NAD + levels result in the failure of oxidative phosphorylation in energy production, leading to reliance on the glycolytic system for ATP production. However, cAMP is required for the activation of phosphofructokinase-1, which is required for the conversion of fructose 6-phosphate to fructose 1,6-bisphosphate in the glycolytic system, so the decrease in cAMP associated with the increased inhibitory serotonin receptors will severely hinder the production of sufficient energy even in the glycolytic system. As expected, ATP production was decreased by GREM2 expression and BMP inhibitors. Reduced NAD + levels and ATP production are markers of cellular senescence, so we confirmed that senescence was induced by BMP signal inhibition.

We further verified the results of this experiment using RNA-sequencing data of large-scale clinical samples, which could be classified into two groups with high and low GREM2 expression. Enrichment analysis using GSEA showed that the expression of genes involved in oxidative phosphorylation and the expression of inhibitory serotonin receptors changed depending on the level of GREM2 expression.

A limitation of this study is that an exhaustive metabolome analysis was not conducted. However, the comprehensive expression analysis showed that GREM2 and BMP inhibitors shifted kynurenine/NAD + production to serotonin production, resulting in cellular senescence and calcification, which is consistent with the results of previous metabolome analyses. Higher serotonin content and lower kynurenine in grade 1 compared to grades 2 and 3 were found by mass spectrometry analysis of meningioma metabolites [30]. Clusters with high expression of tryptophan metabolites, including kynurenine acid, indicated predominantly worse prognosis than clusters with low expression [31], based on detailed mass spectrometry results. These studies support our findings that the proliferative capacity of tumors is altered by a shift in the tryptophan metabolic pathway. Our present study showed that the underlying mechanism by which the metabolite accumulates is mediated by the GREM2/BMP signal-tryptophan metabolism axis, which has not been clarified previously, and that this is directly related to the production capacity of ATP/cAMP. One more limitation is the concentration of K02288. The concentration used in this study is considered to be working on BMPR1A based on the IC50 value, but the possibility of an off-target effect cannot be ruled out. In the future, we believe it is necessary to search for a small molecule with a structure similar to the structural formula of K02288 that acts at a low concentration.

In conclusion, the GREM2/BMP signal controls cellular senescence by shifting tryptophan metabolism and regulating the expression of NAD + /NADH, serotonin, and inhibitory serotonin receptors. The present findings suggest a new target for chemotherapy for recurrent malignant meningiomas.

### Supplementary Information

Below is the link to the electronic supplementary material.

Supplementary file1 (PDF 141 KB)

Supplementary file2 (PDF 158 KB)

## Data Availability

The data discussed in this publication are available in the DDBJ (https://www.ddbj.nig.ac.jp/index.html) under the accession numbers SAMD00642745, SAMD00642746, SAMD00642747, SAMD00642748, SAMD00642749, and SAMD00642750, and GEA under the accession number A-GEOD-22844**.**
