# Supplementary material for: Inhibition of BMP signaling pathway induced senescence and calcification in anaplastic meningioma
Source: J Neurooncol. 2024 Mar 6;167(3):455–65. doi: 10.1007/s11060-024-04625-2 (PMC11096233; doi:10.1007/s11060-024-04625-2)
Supplement: Supplementary file 1 — Supplementary file1 (PDF 141 KB) [file 11060_2024_4625_MOESM1_ESM.pdf]

Inhibition of BMP signaling pathway induced senescence and calcification in anaplastic meningioma

Journal of Neuro-Oncology

Kiyotaka Yokogami,
Department of Neurosurgery, Faculty of Medicine, University of Miyazaki, Miyazaki, Japan

akatoyik@med.miyazaki-u.ac.jp

Supplementary Fig. 1

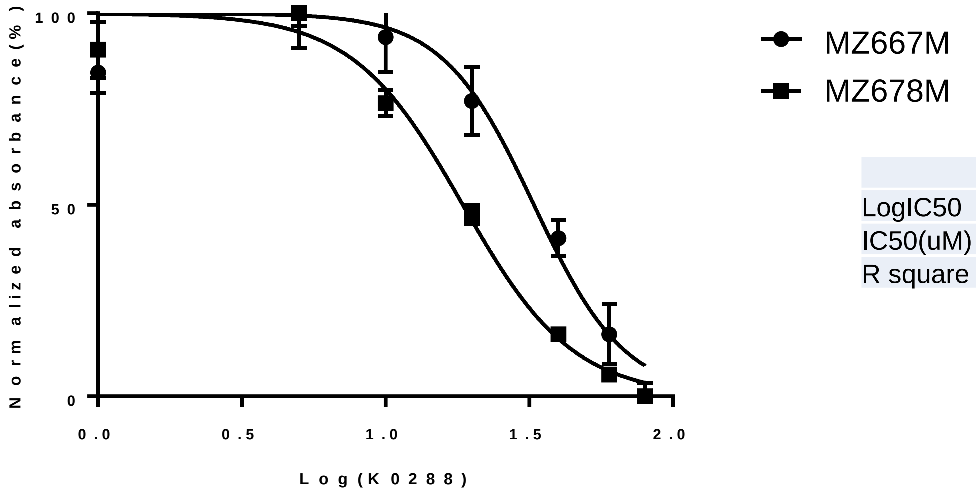

	MZ667M	MZ678M
LogIC50	1.516	1.269
IC50(uM)	32.83	18.59
R square	0.8567	0.9315

The IC50 of K02288 in benign fibrous meniniomas (MZ667M and MZ678M) are32.83 uM and 18.59 uM, respectively.

ALK2	ALK1	ALK6	ALK3
1.1 nM	1.8 nM	6.4 nM	34.4 nM

The IC50 of K02288 against subtype of ALK receptors. (modified from Sanvitale et. al [32])

Supplementary Fig. 2

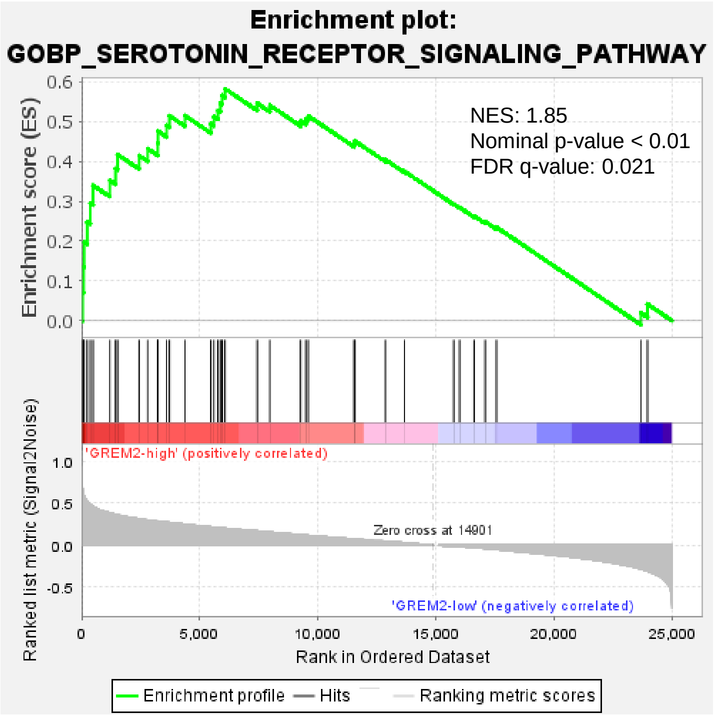

Top10 genes

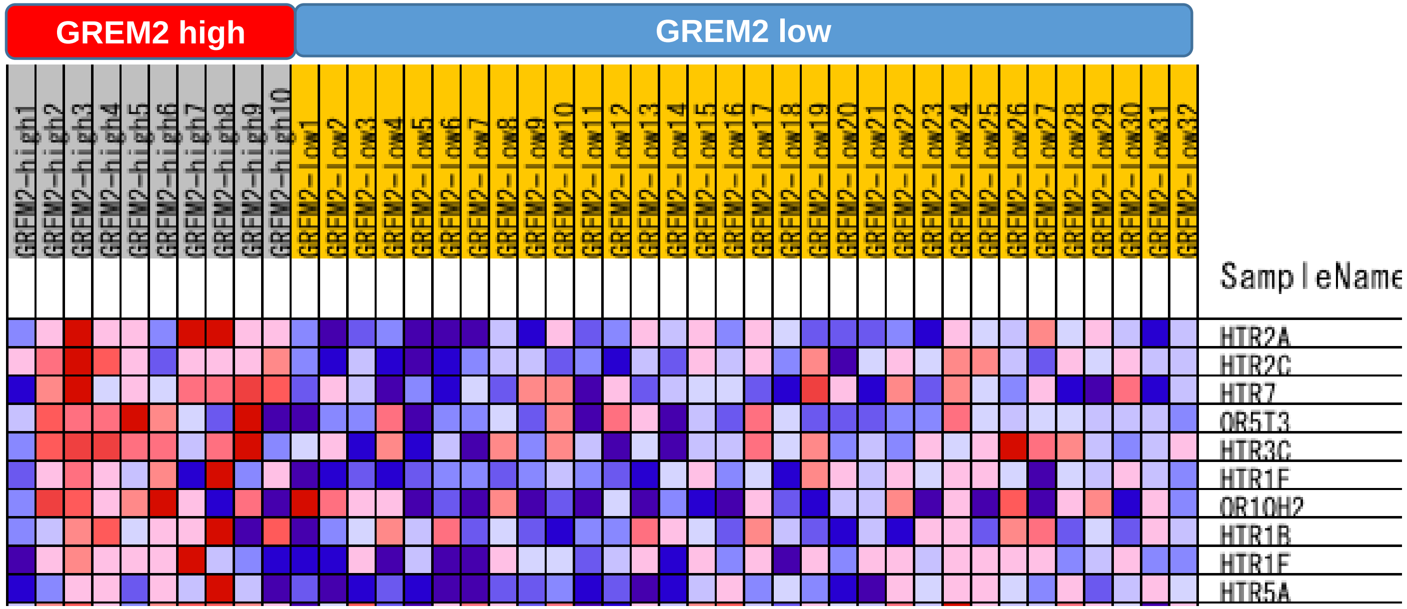

Enrichment analysis using the dataset GSE101638 showed that high expression of serotonin receptors in GREM2 high group.

Supplementary Fig. 3

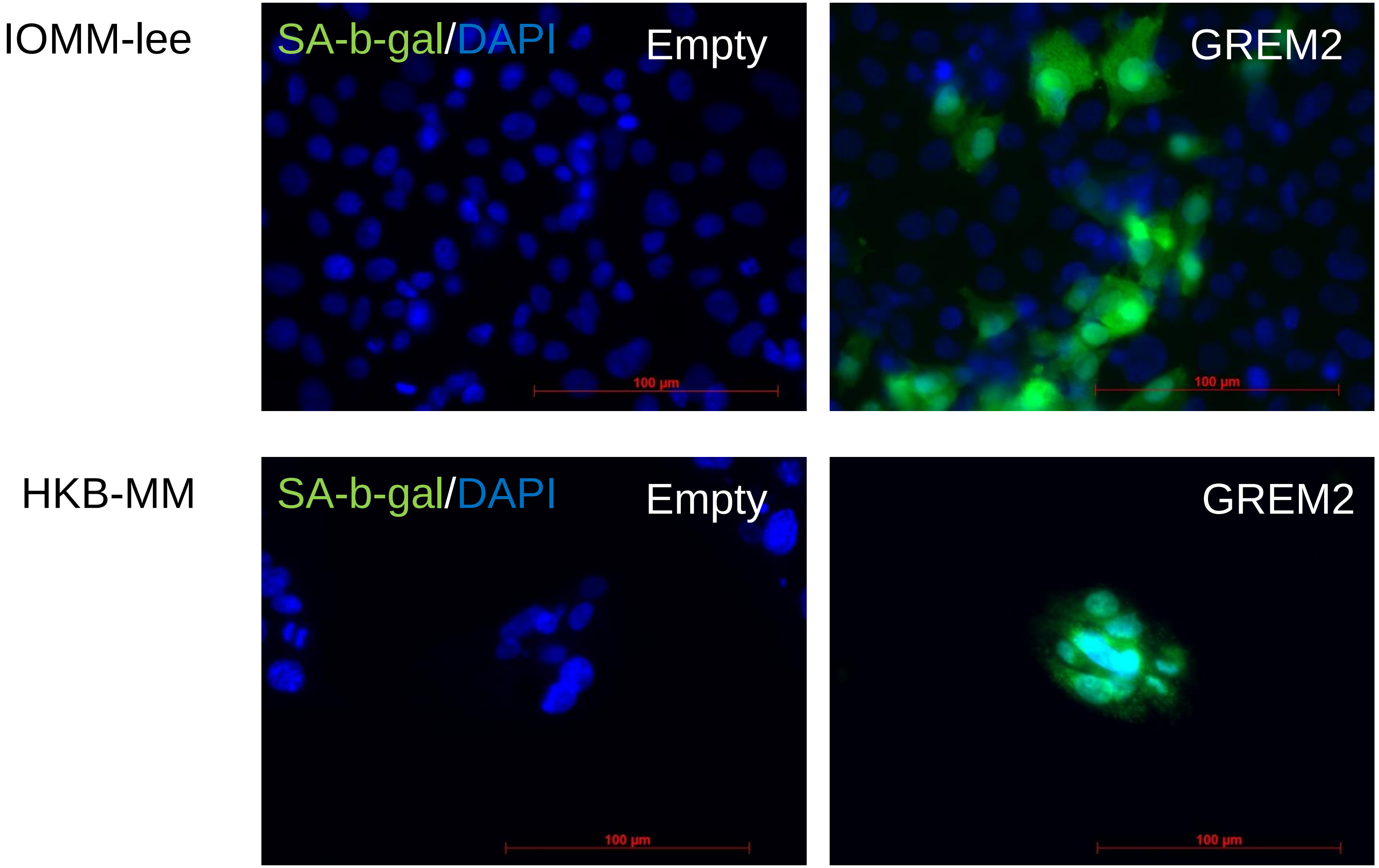

Overexpression of GREM2 induced senescence.
