# Supplementary material for: Inhibition of BMP signaling pathway induced senescence and calcification in anaplastic meningioma
Source: J Neurooncol. 2024 Mar 6;167(3):455–65. doi: 10.1007/s11060-024-04625-2 (PMC11096233; doi:10.1007/s11060-024-04625-2)
Supplement: Supplementary file 2 — Supplementary file2 (PDF 158 KB) [file 11060_2024_4625_MOESM2_ESM.pdf]

Inhibition of BMP signaling pathway induced senescence and calcification in anaplastic meningioma

Journal of Neuro-Oncology

Kiyotaka Yokogami,
Department of Neurosurgery, Faculty of Medicine, University of Miyazaki, Miyazaki, Japan

akatoyik@med.miyazaki-u.ac.jp

Supplementary Table

Character of established cell lines

name	age	sex	pathological diagnosis	MIB-1 L.I.	location
MZ821M	37	F	Transitional meningioma	1.4	tuberculum sellae
MZ840M	58	F	Meningothelial meningioma	5	convexity
MZ842M	77	F	Meningothelial meningioma	1.5	tentorial
MZ857M	74	F	Anaplastic meningioma	50	convexity
